# A Method for Rapid, Quantitative Evaluation of Particle Sorting in Microfluidics Using Basic Cytometry Equipment

**DOI:** 10.3390/mi14040751

**Published:** 2023-03-29

**Authors:** Robert Salomon, Sajad Razavi Bazaz, Wenyan Li, David Gallego-Ortega, Dayong Jin, Majid Ebrahimi Warkiani

**Affiliations:** 1 Institute for Biomedical Materials & Devices (IBMD), Faculty of Science, University of Technology Sydney, Sydney, NSW 2007, Australia; 2 Children’s Cancer Institute, Lowy Cancer Centre, UNSW Sydney, Kensington, NSW 2052, Australia; 3 School of Biomedical Engineering, University of Technology Sydney, Sydney, NSW 2007, Australia

**Keywords:** microfluidics, cytometry, cell sorting, separation

## Abstract

This paper describes, in detail, a method that uses flow cytometry to quantitatively characterise the performance of continuous-flow microfluidic devices designed to separate particles. Whilst simple, this approach overcomes many of the issues with the current commonly utilised methods (high-speed fluorescent imaging, or cell counting via either a hemocytometer or a cell counter), as it can accurately assess device performance even in complex, high concentration mixtures in a way that was previously not possible. Uniquely, this approach takes advantage of pulse processing in flow cytometry to allow quantitation of cell separation efficiencies and resulting sample purities on both single cells as well as cell clusters (such as circulating tumour cell (CTC) clusters). Furthermore, it can readily be combined with cell surface phenotyping to measure separation efficiencies and purities in complex cell mixtures. This method will facilitate the rapid development of a raft of continuous flow microfluidic devices, will be helpful in testing novel separation devices for biologically relevant clusters of cells such as CTC clusters, and will provide a quantitative assessment of device performance in complex samples, which was previously impossible.

## 1. Introduction

The application of continuous-flow microfluidic devices for the separation of cells and particles has expanded in recent years. This has been driven by a broadening recognition that microfluidic devices have significant applications in cell handling, the advent of 3D printers that can be used to produce inertial microfluidic devices [1], the increasing availability of fee-for-service microfluidics device manufacturing [2,3], and biological evidence pointing towards the importance of very rare cells (such as circulating tumour cells (CTCs)) in disease [4].

For those new to microfluidic particle separation and those trying to apply these separation methods to new sample types, validating that a continuous-flow microfluidic device works in your hands and for your samples is critical. This is especially true in cases where events of interest are rare and samples are precious, such as, for example, clinical specimens from cancer patients in the context of using microfluidics for CTC enrichment. The challenge is particularly compounded in clinical specimens as these samples are difficult to assess using standard imaging techniques because of debris and high cell concentrations.

Microfluidic sorting devices can operate by the means of active forces, including acoustic, magnetic, electric, or optic, or by the use of fluid flow characteristics and the microchannel geometry. Given the variety of channel types and the wide range of applications for microfluidic sorting, it is necessary to have a robust, quantitative approach to validate device performance. Traditionally, microfluidic sorting devices have been validated by three approaches––namely, fluorescent tracing of labelled microbeads, high-speed imaging, and the collection of outlets and counting of particles/cells in each outlet. However, all of these approaches suffer from major drawbacks. For instance, these methods may struggle to accurately quantify device behavior when complex and high concentration sample mixtures are used, and many methods are not optimized to evaluate and enumerate the doublets or clusters of cells. More importantly, quantitative assessment of the device performance using the abovementioned methods is often challenging and highly prone to human error. Whilst some articles have used flow cytometry to objectively and quantitively characterise device performance [5,6], for those new to the field, it can be unclear how the characterisation was performed and how to normalise counts and calculate metrics. Therefore, we have developed a step-by-step method using flow cytometry to quantify device performance.

This method provides reproducible, quantitative data on a device’s ability to separate all target cells (a metric we refer to as Separation Efficiency (SE)) as well as the degree to which non-target cells contaminate the target population (a metric commonly known as Purity (P)). This provides a quantitative assessment of particle/cell separation in continuous-flow microfluidic devices. Importantly, because we can perform multiparametric characterisation using flow cytometry, the method described here does not rely on the material used for microfluidic device manufacture, the technique used for the particle manipulation, or the final sample that will ultimately be run. Thus, we provide a universal method to quantitatively assess device performance in any sample in which flow cytometry can be used to identify the population of interest.

## 2. Statement of Purpose

This paper describes a method for quantitatively assessing the performance of continuous-flow microfluidic sorting devices in complex systems. We show how device performance can be measured using complex mixtures, including those that contain beads alone and those that contain both beads and cells. We describe, in detail, how to perform simultaneous quantitative assessment in an example device that separates particles and cells based on size, and we show how we can use this method to assess the behaviour of multiple cell/particle sizes within a single experiment. Furthermore, we show that this method performs in conditions that are impossible to accurately quantitate using commonly employed imaging methods, and we describe how flow cytometry-based pulse processing can be used to quantitively assess the behaviour of cell and particle clusters. As all this is achieved using readily available instruments, we expect this method to become a widely used and cost-effective approach for validating continuous-flow microfluidic devices for particle sorting.

## 3. Methods

### 3.1. Overview

Beginning with step 1, sample preparation, the first step is to create a test sample containing a variety of different-sized particles and/or cells. Following this, we move to step 2, which is microfluidic separation, when, during microfluidic separation, the sample is run through the microfluidic channels to perform particle separation. Following this, we move to step 3, sample collection and processing. As part of this procedure, samples are collected into their own collection tubes and measured so that outlet volumes can be made equal and count beads can be added. This creates samples that can be volumetrically measured on a standard cytometer, even when volumetric sample acquisition is not provided on the cytometer. Once this normalisation process has been completed, we move to step 4, flow data acquisition, which is a step that has been designed to work using basic flow cytometry equipment and only requires that appropriate gain settings be used so that individual populations of interest can be readily gated during step 5 data analysis. Following gating of flow data, we move to step 6 in which the metrics for SE and P are calculated. During this step, data exported from FlowJo is analysed using a combination of Excel and RStudio to produce a quantitative assessment of device performance.

**Figure 1:**
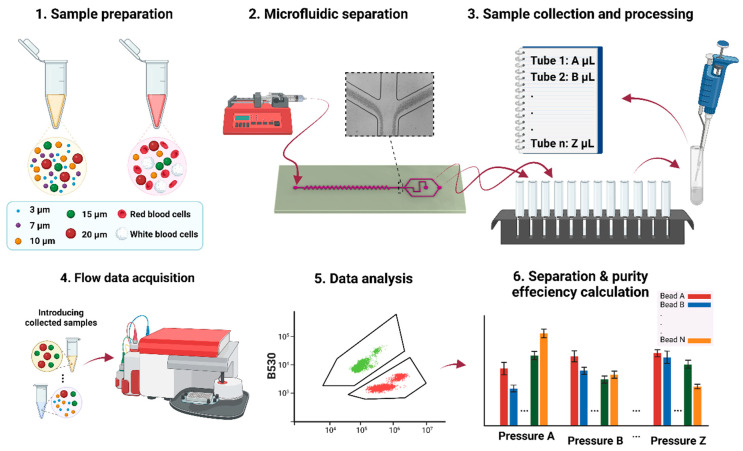
Overview of the method to quantitatively assess the performance of microfluidic sorting devices using flow cytometry. This method is a 6-step process involving sample preparation, microfluidics separation, sample collection and processing, flow data acquisition, data analysis, and the calculation of the quantitative metrics. Together, this allows a robust and quantitative way to assess the performance of any continuous flow microfluidic device that enables particle separation.

By following these steps, we provide an end-to-end protocol that quantitatively assesses not only device performance in relation to particles of different sizes but also clusters of beads/cells. The order in which these are performed is shown in Figure 1.

### 3.2. Detailed Methods

**Step 1: Sample preparation**

This method works equally well with bead mixtures as it does with cell mixtures. When assessing the performance of new devices, we recommend using beads as these are a readily available and renewable sample that provides high signal-to-noise ratios leading to a simpler gating strategy in flow cytometry analysis. Cell mixtures, on the other hand, are often employed as a secondary validation step. Cell mixtures are critical in biological applications as cells may behave differently from beads and, thus, it is imperative that the device is tested in a situation as close as possible to real-world test conditions.

1a Bead mixtures: To ensure that individual bead populations can be identified by flow cytometry, we initially ran a large batch of beads through the cytometer (BD FACSAria III or BD S6 cell sorter, both from (BD Biosciences, San Jose, CA, USA)). From these results, we were able to select individual bead populations that (1) represent particles of a size commonly found in blood, (2) could be accurately separated from each other (using signals from both the scatter and fluorescent parameters), and (3) did not form large clumps (that could block channels). Results from this preliminary analysis identified suitable beads of 3 μm, 7 μm, 10 μm, 15 μm, and 20 μm. Bead stocks were then counted using a standard haemocytometer so that a bead solution could be created to test device behaviour with suspensions of the individual-sized bead at concentrations reflective of those found in biology (i.e., larger particles representing CTCs spiked in at a concentration much lower than the 7 μm and 10 μm particles that represent red blood cells (RBCs) and white blood cells (WBCs), respectively). Final bead suspensions were made in Phosphate-buffered saline (PBS) (ThermoFisher Scientific, Sydney, Australia) with 5% Foetal Calf Serum (FCS) (ThermoFisher Scientific, Sydney, Australia) at the concentrations shown in Table 1.

1b Beads in blood mixture: Blood (obtained, with informed research consent, through an agreement with the Australian Red Cross Blood Bank) was first stained with CD45 (1:200) and then diluted (1:20) with PBS containing 5% FCS. The concentration of the 10 μm, 15 μm, and 20 μm beads that were spiked in was the same as that shown in Table 1.

**Step 2: Microfluidic separation**

Device setup: Microfluidic devices require precisely controlled flow rates to perform optimally. This can be achieved using either syringe pumps, controlled pressure delivery systems, or pressure delivery systems that utilise flow rate sensors to actively adjust pressures according to flow rates. The method described here works with both Syringe Pumps (i.e., Chemyx Fusion 200, Chemyx, TX, USA) and pressure-driven sample delivery (i.e., Fluigent Line-up Flow EZ 2000, Paris, France). As syringe pumps are cheaper and arguably more common, especially in labs just beginning to interface with microfluidics, we only show data from syringe-driven separations. In this article, each experiment has been repeated three times in three different days with a new microchannel and batch of beads.

High-speed imaging data acquisition (optional) involved high-speed imaging that was achieved using the phantom high-speed camera (PHANTOM VEO-E 340L MONO, Vision Research, Wayne, NJ, USA) mounted on an inverted microscope (Olympus IX-83, Tokyo, Japan). The acquisition was achieved using Phantom Camera Control (PCC, version 3.5).

**Step 3: Sample collection and processing**

After stabilisation of the flow rate, i.e., no bubbles were being pushed through the system and the device appeared to be performing appropriately by visual inspection under a microscope, outlet tubes were placed into the collection tubes at the exact same time. Following the collection of ~150 μL in the lowest volume tube, both outlet tubes were first returned to the waste collection, and the pressure stopped. When performing outlet collection, it is important to ensure that (1) the device has been running stably for long enough for the sample within the collection tubing to accurately reflect device performance (this is so that the sample collected from the outlet tubing is obtained from the time in which the device was functioning correctly); (2) both fractions are collected for the same length of time; and (3) the collection tubing is returned to the waste tube prior to stopping the sample delivery or running out of the sample.

Following the collection, the volume of the sample must be determined. This can be achieved by weighing the sample or measuring it with a calibrated pipette. Once the volume of each collection tube is known, the collection tube containing the lowest volume for each run (in devices tested here, this is the inner or central fraction) is topped up with buffer to ensure both fractions contain the same volume. As a control step, the fraction of inner tube volume to total volume can be calculated. In the devices characterised here, the fraction remains the same within the flow rates tested, and approximately 33% of the sample is collected in the inner channel while 66% flows through the outer channels. Therefore, tubes with altered collection ratios can be excluded from further analysis as this suggests an outlier in the collection process.

Following this, an equal volume of count beads was spiked into each sample. In this case, we used accudrop beads (BD Biosciences); however, any beads/particles that can be accurately separated from the beads/cells being tested can be used.

**Step 4: Flow data acquisition**

Data was analysed on a standard cytometer; we used the BD FACS Aria III or BD S6 cell sorter. However, as there are no unique instrument requirements, any instrument would work. Voltages were set by running single stain controls, and forward scatter (FSC) area and height, as well as side scatter (SSC) area and height, were collected to allow doublet/cluster analysis.

**Step 5: Flow data analysis**

Data was exported as FCS 3.0 or above and analysed with FlowJo software (version 10.8.1). Gates were set on single stained controls with careful positioning to ensure gates encompassed most of the target population without overlapping with other populations. For sized-based bead determination, we found that the fluorescence signal better separated the particle size and that a combination of gates could accurately distinguish all populations within the complex mixture.

**Step 6: Calculating quantitative performance**

With the ability to accurately separate each individual bead and cell population, we then performed a count correction based on the volume of samples collected during the recording period. Briefly, as we topped up the two collection tubes to the same volume by adding the same number of accudrop beads to each tube, we could determine the relative volume of each tube recorded during data acquisition. For example, suppose 10,000 accudrop were recorded in the inner tube and only 5000 were recorded in the outer tube. In that case, the number of cells and beads collected in the inner tube should be doubled to correct for different acquisition volumes. Specifically, the accudrop bead numbers in both collection tubes were each divided by the accudrop bead numbers in the tube that collected the non-target population, and, in our case, this was the outer outlet. We then multiplied the counts from each bead population by this number to obtain normalised cell/particle counts.

After correcting gated populations according to the acquired sample collection ratio (based on accudrop bead spike in numbers), we determined the performance of the devices based on two metrics:

Separation Efficiency (SE): measures the fraction of the target population collected in the desired outlet. It can be calculated as:

(1) $$\mathrm{SE}=\frac{corrected\;target\;population\;count\;in\;target\;outlet\;}{Corrected\;target\;popultaion\;count\;in\;target\;outlet+target\;population\;count\;in\;other\;outlet\left(s\right)}\times 100$$

Purity (P): measures how many non-target events are contained in the same collection fraction. This can also be obtained directly from the flow acquisition file using the Freq. total statistic in FlowJo.

(2) $$P=\frac{count\;of\;target\;population\;in\;target\;outlet\;}{count\;of\;nontarget\;popualtion\;in\;target\;outlet}$$

Final data analysis and graphical representation of the results were performed in RStudio.

## 4. Results and Discussion

Here we show that this method can be used to quantitatively assess the performance of continuous flow microfluidic devices using both a bead-only sample and a bead mixture based on diluted whole blood to model the performance of real cells within the device. This is an improvement on the current methods of fluorescent tracing, high-speed imaging, and image-based particle counting, and it allows the analysis of complex mixtures where other methods would fail.

### 4.1. Gating Strategy for Bead Populations

**Figure 2:**
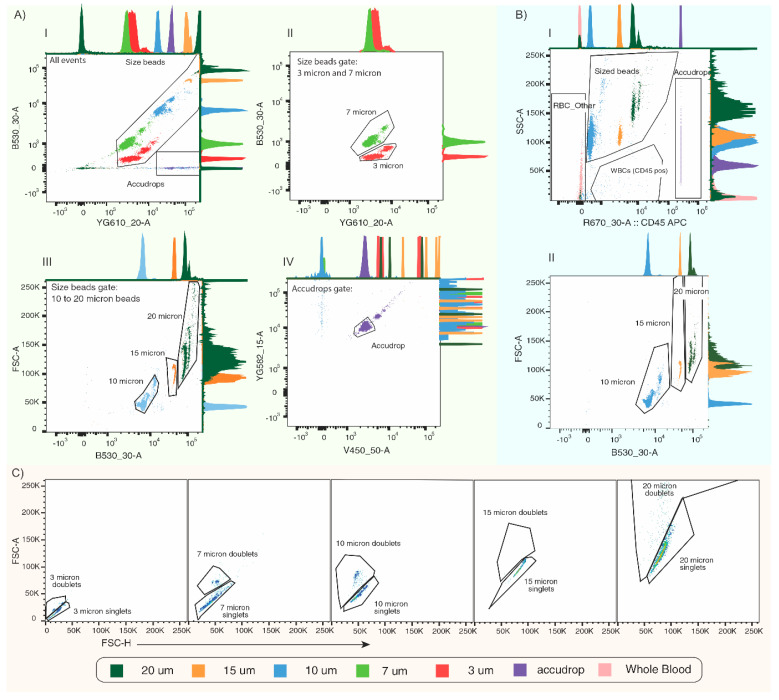
Gating strategy for the beads and cell samples used to validate the method. (**A**) gating strategy for bead only mix, (**I**) shows the initial separation of size beads from the counting beads (accudrops), (**II**,**III**) illustrates the gating method used to separate 3 μm, 7 μm, 10 μm, 15 μm, and 20 μm beads from each other, (**IV**) shows the gate used to further identify the accudrop beads used for volume correction. (**B**) Gating strategy for blood and bead mixture (**I**) shows the initial separation of beads, cells and count beads, and (**II**) illustrates the separation of 10 μm, 15 μm, and 20 μm beads from each other. (**C**) Doublet discrimination/cluster gating for 3 μm, 7 μm, 10 μm, 15 μm, and 20 μm beads.

To allow the quantification of individual populations within complex mixtures, developing a gating scheme that allows minimal overlap between populations is necessary. If individual populations of interest can be separately gated without overlap, it does not matter how population separation is achieved. In this example, we show a gating strategy that works for both the beads mixtures alone and for beads spiked into diluted whole blood samples. Bead mixtures containing a mixture of fluorescently labelled beads at 3 μm, 7 μm, 10 μm, 15 μm, and 20 μm can be separated based on a combination of fluorescent channels, as shown in Figure 2. The B530 fluorescent channel is linked to excitation via a 488 nm laser with the detector restricted by a 488 long pass and a 530/30 bandpass filter. This was matched to the maximum excitation/emission of the beads used in our sample. Used alone, it allowed reasonably good separation of beads, but due to signal overlap of the 3 μm doublets and the 7 μm singlets in this channel, we also used the YG610 to assist in separating particles. The accudrop beads (used to correct for volume acquisition during file recording) are also detected in this channel and could be gated based on the high B610 emission (Figure 2(AI)).

We also looked at separating beads by FSC. While FSC does increase with bead size, the overlap between bead sizes prevents separation; however, by combining FSC and B530 signal, beads of sizes 10 μm, 15 μm, and 20 μm could be separated (Figure 2(AIII)). To complete the separation, we gated the 3 μm doublets from the 7 μm singlets beads based on differential signals in the YG610 channel (Figure 2(AII)). Figure 2(AIV) shows the final clean-up gates allowing accurate counting of accudrop beads.

To separate bead populations from whole blood, we found that size beads, RBCs, WBCs, and accudrop beads could be separated using a combination R670 (which stained for CD45 using APC) and Side Scatter (Figure 2(BI)). From there, the size beads containing 10 μm, 15 μm, and 20 μm beads could be separated using the B530 channel (Figure 2(BII)).

Finally, we show that pulse processing of the height and area signals collected from the forward scatter on the gated bead population can be used to identify single beads and those that travel as doublet (Figure 2C).

### 4.2. Quantitative Characterisation Using Bead Populations to Identify Appropriate System Conditions

To simulate the characterisation process of a new device, we first ran a mixed bead population (3 μm, 7 μm, 10 μm, 15 μm, and 20 μm) through a variety of chips made from both PDMS and 3D printing. We used both pressure delivery and volume delivery on both chip types and concluded that neither chip type nor the mode of sample delivery affected the ability of our approach to quantify device performance.

As an example of the method, we applied it to quantitively assess the performance of 3D printed chip using zigzag geometries that we have previously published [7]. The active mechanism of this device is inertial focusing and achieves the separation based on size. In many cancers such as breast [8,9], lung [9,10], glioblastoma [11], and prostate cancer [12], CTCs tend to be larger than “healthy cells” normally found in the blood. To achieve optimal separation of the CTC/CTC cluster, a balance between the recovery of target cells and depletion of non-target cells is desired.

**Figure 3:**
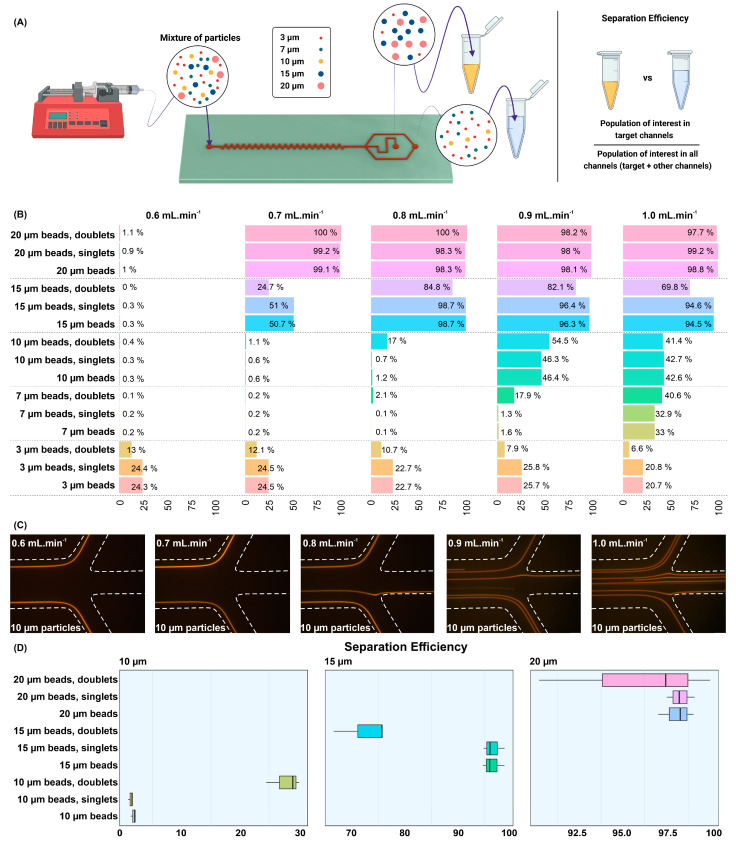
Result from a flow rate titration on a 3D printed inertial focusing device. (**A**) Shows the system setup and a visual representation of how samples are collected, and calculations of SE were made for the central channel, which, in this case, is designed to enrich cells/particles that are above ~12 um. (**B**) Shows the SE as calculated using the gating method shown in Figure 2 for all bead populations tested at different flow rates. Shown here, also, are the SE values for the singlet and doublet events for each bead size. (**C**) Shows the results from a traditional fluorescence trace experiment. These results clearly indicated that (i) the result from each method is similar, the fluorescent trace analysis only shows performance at a single point in time, there is no way to quantify the results based on fluorescent streak analysis, and it is not possible to analyze the behavior of doublets or cluster using fluorescent imaging. (**D**) Shows the reproducibility of our method to calculate SE over three independent experiments.

The quantitative results for flow rate together with the more traditional fluorescent streak imaging, are shown in Figure 3. Figure 3A shows the device setup and highlights how the two outlets are used to determine the SE.

Figure 3B shows how, from a single experiment, it is possible to determine how effectively cells of different sizes can be focused into the central collection channel. The SE is shown for each particle size, and the doublet/cluster associated with the individual-sized bead. With the device tested, a flow rate of 0.6 mL·min^−1^ will result in none of the larger particles (>15 μm) being focused to the central channel. Therefore, we can conclude that for this device, the flow rate of 0.6 mL·min^−1^ will not achieve the goal of enriching larger cells/CTCs and therefore cannot be used. At 0.7 mL·min^−1^, nearly 100% of 20 μm particles are found to be focused into the central channel; however, as only 50.7% of 15 μm beads and 24.7% of 15 μm doublets are collected, this flow rate is not appropriate either. When we move to 0.8 mL·min^−1^ and above, a capture rate of nearly 100% of 20 μm particles and more than 98% of 15 μm beads is achieved in the central outlet. Interestingly, the capture of 15 μm doublets/clusters peaks at 0.9 mL·min^−1^, and whilst this flow rate will give the optimal recovery of potential CTCs and CTC clusters, the collection of 10 μm particles (which are not the desired population of interest) dramatically increases (46.3% of 10 μm singlets and 54.5% of 10 μm doublets/clusters are also captured). Therefore, the optimal flow rate for the device tested is 0.8 mL·min^−1^ as it balances the capture of large particles and their doublets whilst minimising contamination of smaller particles.

Figure 3C shows images taken using the traditional method of microfluidic device testing, whereby high-speed imaging or fluorescent imaging is used on single populations of beads to assess device performance. These images highlight the limitations of image-based analysis when using complex mixtures as they cannot provide a quantitative assessment of device performance.

Having used our method to confirm the optimal flow rates on a novel device, we characterised device performance at the optimal flow rates of 0.8 mL·min^−1^ over three independent experiments. Figure 3D highlights the reproducibility of the method and reflects the expected device performance based on traditional modes of device validation.

Our results are in line with the behaviour seen in imaging but can be performed in complex mixtures (not just single bead populations) and provide quantitative data for both Purity and Separation Efficiency.

### 4.3. Quantitative Characterisation Using Beads and Blood

Whilst characterisation using beads is an important first step in validating device performance, devices designed to be used in blood samples should be validated using blood. Blood is a complex biofluid that can exhibit traits of both a Newtonian and non-Newtonian fluid [13]. Furthermore, it consists of high particle concentrations that are near impossible to replicate using beads. For example, blood with a haematocrit of 40–45% will contain approximately 4.8 to 5.31 billion (10^9^) RBCs, 5.8 to 7.44 million (10^6^) WBCs, and 230 to 285 million (10^6^) platelets per mL [14]. Whilst some studies have used 7% Ficoll PM 70 to simulate the viscosity of a whole blood when diluted 1:1 [15], this would not allow calculation of the Purity metric.

As such, rather than trying to replicate the concentration of RBCs with 3 μm and 7um, healthy donor blood can be spiked with beads greater than 10 μm to represent CTC behaviour. To simulate the behaviour of large particles in complex fluids such as blood, we spiked beads of size 10 μm, 15 μm, and 20 μm into diluted blood and showed that device behaviour could still be quantified using our method.

**Figure 4:**
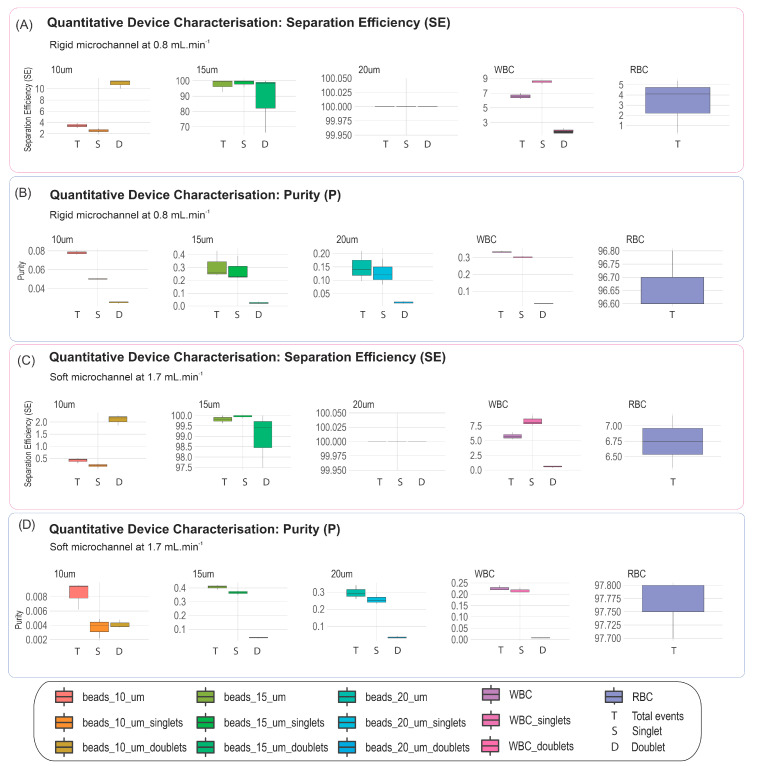
Quantitative assessment of two different devices run at their optimal operational flow rates. SE and P values are calculated for three independent replicates of sample composed of diluted whole blood spiked with 10 μm, 15 μm, and 20 μm beads. (**A**,**B**) show the SE and P values for a 3D printed chip, while (**C**,**D**) show the SE and P values for the PDMS spiral chip. Values are calculated for each particle/cell type and have been split by total (T) events for that particle/cell type, singlets (S), or doublets (D).

The quantitative results are shown (Figure 4) for a 3D printed and a PDMS (spiral geometry). These have been optimised to work at flow rates of 0.8 mL·min^−1^ and 1.7 mL·min^−1^, respectively, and we thus assess the robustness of this method by assessing device performance in triplicate.

Figure 4A shows the SE for a novel 3D printed chip (the fundamental behaviour of particle focusing within this channel can be found in our previous publication [7]) while Figure 4C shows the SE using a previously published PDMS chip [8].

Both chips are designed to enrich for cells larger than 12–15 μm and, as we are comparing cell counts from the target channel to those in the other channel results, should show high SE values for 15 and 20 μm and low SE values for 10 μm beads. In Figure 4A, which compares the SE for each particle in the Zigzag, results show that the chip provides excellent SE values for 15 μm and 20 μm beads. Combined with the low SE values of approximately 4%, 7%, and 4% for 10 μm beads, WBCs, and RBCs, respectively, we can see that the chip is effective in focusing large particles to the inner channel whilst removing smaller particles, including WBCs and RBCs. Importantly, we can track the SE values for all particles of one type and separate these to further provide an SE value of doublets/clusters and of singlets. The ability to do this is highlighted when we compare the behaviour of doublets (denoted by the column D in the graphs) between the Zigzag and the spiral ships. The Zigzag chip is designed to focus doublets and, thus, one would expect more 10 μm bead doublets in the Zigzag chip. Results indicate that >10% of 10 μm doublets are focused on the collection channel for the Zigzag chip (Figure 4A), while this figure was only ~2% for the Spiral device (Figure 4C). This shows a 5-fold improvement in the ability to focus 10 μm bead doublets when moving from the Spiral device to the Zigzag device.

As these devices were tuned to prioritise recovery of rare events over absolute purity, it is reasonable to expect some contamination. The high Purity (P) of RBCs in both chips of ~97% (Figure 4B,D) indicates just how difficult it is to enrich for particles of interest when they are present at extremely low rates in the original sample.

Despite choosing an extremely challenging situation (diluted whole blood), this method still shows that quantitative assessment of particle behaviour in a complex system is indeed possible. Importantly, these results show our method is capable of robustly and quantifiably assessing device performance (P and SE values) where other systems (such as imaging) would be overcome by noise. In this example, we are able to reproducibly count a single 15 μm bead amongst approximately 300 other events and approximately one single 20 μm bead for every 650 contaminating particles.

There are several applications where continuous-flow microfluidic devices can be used to achieve practical outcomes. These can be quite varied, and range from bacteria separation to prevent beer spoilage [16], exosome subpopulation separation [17], neutrophil purification in diabetes [18], separation of cycling cells [19], dead cell removal [20], and enrichment of CTCs [21] to be used for further multiomics data analysis using single cell methods [22,23]. Additionally, the discovery that CTC clusters play an important role in the metastatic cascade [24,25,26,27,28,29] has created a need to develop devices that can enrich CTC clusters. For further information regarding the relevance of this method to CTC and CTC cluster enrichment, see Appendix A.

Regardless of the situation in which continuous-flow microfluidics sorting is used, the ability to quantifiably analyze particle behaviour in complex biological mixtures (such as beads spiked into blood) is important as it allows an accurate assessment of device performance and ultimately gives confidence that precious clinical samples can be run without fear of the device not performing as expected. Table 2 has been provided, comparing all the methods developed to characterize microfluidic devices for particle and cell sorting.

## 5. Conclusions

Our method is a significant advancement on the current status-quo for testing continuous flow microfluidic devices as it allows the analysis of complex mixtures and expedites the understanding of how particles and cells behave within the chip. This method is superior to existing image-based approaches as it:

1. Provides quantitative data on two key metrics of device performance, namely:
  a. Purity
  b. Separation Efficiency
2. Allows the simultaneous analysis of
  a. Multiple particles
  b. Complex cell mixtures
3. Can distinguish between doublets and clusters even when these are exceedingly rare events

The results shown highlight the power of the method, the use of which will substantially reduce device testing times and will give confidence that the results produced are not only highly reproducible but also quantitative. By using equipment regularly found within a flow cytometry facility, this protocol should be able to be implemented by most labs and will facilitate a rapid, quantitative assessment of how emerging microfluidic devices behave with specific cell types and conditions. Furthermore, it is a quick and low-cost method that does not rely on the expensive high-speed cameras often only found in specialist labs.

## Figures and Tables

**Table 1:** Bead mixture used to validate the method consisted of beads selected from 3 μm to 20 μm that could be accurately resolved from each other using standard fluorescent flow cytometry. Full details to recreate the samples used in the development of this method are provided.

Bead Size	Cat Number	Volume Added to 15 mL PBS +5% FCS	Estimated Concentration
3 μm	CAF-003UM	100	4.13 × 10^6^/mL
7 μm	CAF-007UM	30	286 × 10^3^/mL
10 μm	CAF-010UM	40	161 × 10^3^/mL
15 μm	PM015UM	60	51 × 10^3^/mL
20 μm	PM020UM	120	25 × 10^3^/mL

**Table 2:** A comparison of approaches used for evaluation of microfluidic devices utilized for particle and cell sorting.

Method	Approach Summary	Comments
Haematocytometer	Measure the total sample volume, collect 10 –20µL of the sample, use a haematocytometer to count the particles or cells, and then characterize the device performance	Advantages: relatively easy; cheap. Disadvantages: Time-consuming; prone to human error; highly dependent on the individual user (although automated counters reduce operator bias); prone to a batch-to-batch difference in the results; not a perfect quantitative approach for device performance; not suitable for rare cells or clusters; not ideal for samples with high concentration or with a mixture of cells or particles.
High-speed camera	Record device perfomracne using a large number of frames per second and visually inspect performance.	Advantages: Can provide the motion of particles or cells passing through the channels; can distinguish between cells and clusters. Disadvantages: Only takes a very short snapshot of device behaviour (in the order of µseconds); not recommended for rare cells; high-speed cameras are rare outside of specialist microfluidic labs; and requires high sampling rates, large storage volumes, and advanced image analysis and devices in which the depth of the channel does not exceed the depth of the focal plane in order to calculate metrics for particle or basic cell separation efficiency or purity. In the case of complex cell mixtures, it would require the development of novel label free cell classification algorithms as well as the above considerations for particles and basic cell separation in order to calculate device performance metrics.
Fluorescent tracing	Evaluate the device performance using fluorescent trace of particles or cells	Advantages: Can illustrate the trace of fluorescent particles passing through the channel. Disadvantages: Restricted for analysis of highly fluorescent microbeads; only applicable for brightly stained cells/particles cells; unable to provide metrics for separation efficiency or purity; cannot distinguish between single or clusters of particles; cannot be used with mixture of particles with different sizes; only takes a snapshot from device performance over a very short period of time.
Current study	The step-by-step protocol has been provided in Section 3.2 of the article	Advantages: Highly reproducible; independent of the user; provide metrics for device performance (i.e., separation efficiency or purity); ideal for rare cells; can be used with mixture of cells or particles of different sizes; applicable even in high concentration mixture of particles; able to identify the behaviour of doublets or cluster of cells; evaluates the average device performance over a any period of time; as the method uses a cytometer, it enables a variety of ways for characterization and illustration of the device performance; independent of the material used for the microchannel fabrication or volume of the sample. Disadvantages: Requires the use of a flow cytometer as opposed to high speed imaging and fluorescent tracing and does add an extra validation step; best suited to conditions in which cell/particle types can be easily distinguished from each other; and in cases where cell types of interest are very similar, may require high dimensional characterisation panels to calculate metrics.

## Data Availability

The data presented in this study are available on request from the corresponding author.

## Appendix A. Case Study-Enrichment of Ultra-Rare CTCs and CTC Clusters Using Continuous Flow Devices

### Appendix A.1. Biology of CTCs

CTCs are cancer cells responsible for metastatic spread, and have been correlated to disease outcomes in breast cancer [30,31], prostate cancer [32], and colon cancer [33]. The role of CTCs in the lung is less clear, but CTC levels at baseline have been indicated as a negative prognostic factor [34,35]. CTCs are often found circulating within the peripheral blood but can also be found in a variety of body fluids, including cerebral spinal fluid [36,37,38], bone marrow [39,40], and ascites [41].

CTC Clusters are clusters of cells that contain at least one CTC. CTC clusters may be composed of CTCs alone or contain both CTC and other non-cancer cells. Despite being even rarer than CTCs, the identification and characterisation of CTC clusters is important in understanding cancer progression and spread, and there are multiple microfluidic devices that have been designed to allow their separation [15,28,42].

### Appendix A.2. Problem of Isolating CTCs

Both CTCs and CTC clusters are expected to be ultra-rare (clinically relevant at levels above 1 in a billion blood cells). In cancers such as breast, prostate and lung, CTCs have been enriched based on size using inertial microfluidics or via cell surface expression using immunoaffinity methods.

### Appendix A.3. Relevance of Method

Traditionally, CTC enrichment devices have been characterised by running beads through a chip and combining high-speed imaging and fluorescent imaging to look for fluorescent traces (as shown in Figure 3C). However, these approaches do not allow multiple bead sizes to be analysed simultaneously, and nor do they allow quantitative analysis of device performance. Additionally, they struggle to characterise device performance in relation to cluster separation.

Furthermore, when devices are tested using cells, the complexity of the mixtures affects the ability to resolve individual cells/particles via high-speed imaging and as blood cells do not express high levels of fluorescence, they cannot be analysed by fluorescence imaging.

Our method is ideally suited to quantitative analysis of rare cell enrichment approaches as it relies on high throughput flow cytometry. Thus, this method can simultaneously identify the ratio of any population in each collection outlet regardless of how rare the populations are or how difficult they are to characterise using traditional imaging techniques.
